# Predators Are Attracted to the Olfactory Signals of Prey

**DOI:** 10.1371/journal.pone.0013114

**Published:** 2010-09-30

**Authors:** Nelika K. Hughes, Catherine J. Price, Peter B. Banks

**Affiliations:** Evolution and Ecology Research Centre, School of Biological, Earth and Environmental Sciences, University of New South Wales, Sydney, New South Wales, Australia; University of Bristol, United Kingdom

## Abstract

**Background:**

Predator attraction to prey social signals can force prey to trade-off the social imperatives to communicate against the profound effect of predation on their future fitness. These tradeoffs underlie theories on the design and evolution of conspecific signalling systems and have received much attention in visual and acoustic signalling modes. Yet while most territorial mammals communicate using olfactory signals and olfactory hunting is widespread in predators, evidence for the attraction of predators to prey olfactory signals under field conditions is lacking.

**Methodology/Principal Findings:**

To redress this fundamental issue, we examined the attraction of free-roaming predators to discrete patches of scents collected from groups of two and six adult, male house mice, *Mus domesticus*, which primarily communicate through olfaction. Olfactorily-hunting predators were rapidly attracted to mouse scent signals, visiting mouse scented locations sooner, and in greater number, than control locations. There were no effects of signal concentration on predator attraction to their prey's signals.

**Conclusions/Significance:**

This implies that communication will be costly if conspecific receivers and eavesdropping predators are simultaneously attracted to a signal. Significantly, our results also suggest that receivers may be at greater risk of predation when communicating than signallers, as receivers must visit risky patches of scent to perform their half of the communication equation, while signallers need not.

## Introduction

The arms race in tactics of detection and evasion between predators and their prey has profoundly affected the life-history and behavioural strategies adopted by both parties [1]. Arguably, the most fundamental component of this interaction is the means by which predator and prey detect one another, even before any real encounter has begun. Consequently, there should be strong selective pressure on the ability to exploit the residual signs of enemy activity, especially those which may improve early detection or evasion for either player. Any action by predator or prey, whether foraging, signalling for mates or even staying still, has the potential to leave residual evidence of presence that is open to exploitation. For many species, visual and auditory cues are sources of information for immediate use, which has given rise to many strategies of crypsis for both predator and prey [2]. But for other species, especially mammals, the greatest betrayal probably comes from their smell.

All individuals emit odours as the inevitable consequence of metabolic processes combined with genetic individuality, hormonal fluxes, and microbial activity. These odours may be deposited in the environment unavoidably, for example as volatiles released from the body surface into the atmosphere, or deposited onto the substrate via footprints [3]. Distinctive, individual scents are also used for communication with conspecifics. In many mammals, scents (urine, faeces or other secretions; hereafter scent marks) are deliberately deposited onto the substrate (e.g. ground, plant material) and function in mate choice, individual recognition and territorial defence [3]. These scent marks are designed to persist, to provide information to many possible receivers. But this longevity in the absence of the signalling individual means that scent marks are part of an open signalling system that is vulnerable to eavesdropping by individuals other than the intended receiver, both conspecifics and heterospecifics. Examples abound of prey that use the scents of their olfactorily- signalling predators to assess their level of predation risk and respond accordingly [4]. But while predators are known to be attracted to general food [5] and prey cues [6], [7], [8], rarely has the exploitation of deliberate prey scent signals by predators been demonstrated.

For mammalian systems, only three studies have looked at predator attraction to prey scents. Cushing [9] showed that weasels (*Mustela nivalis*) prefer the scents of estrous over diestrous prairie deer mice (*Peromyscus maniculatus bairdi*), and Sundell *et al*. [10] and Ylönen *et al*. [11] demonstrated a similar attraction by weasels to the odours of two common prey species, the bank (*Myodes glareous*) and field (*Microtus agrestis*) vole. All three studies were conducted in small, relatively enclosed areas and controlled conditions where predator behaviour was focussed on the experimental trials. Consequently, the potential risks that prey scent pose in the wild remain largely assumed but unknown [12], [13], [14], and in some cases contested [15].

There is evidence, however, that olfactorily communicating species are sensitive to increased risks of predation when receiving olfactory social signals. Male house mice (*Mus domesticus*), for example, trade-off the social benefits of receiving signals with the perceived risk of predation: mice reduce receiving of low social value signals when the perceived risk of predation is high, while still maintaining rates of receiving of high social value signals [16]. Similar trade-offs have been demonstrated in field voles, who reduce olfactory eavesdropping on a heterospecific competitors scents under an elevated risk of predation [17].

Here we examine whether the scents of a prolific scent marker, the house mouse, are attractive to olfactorily hunting predators. Mice use scent marks in the form of small smears of urine for territorial advertisement, mate choice and individual identification within their highly complex social networks [18]. Marks are overwhelmingly deposited by dominant males, and are generally concentrated around valuable resources and territorial boundaries [18]. Importantly, however, marks are investigated to a similar extent by all individuals; receiving typically requires direct contact with the scent mark [18].

If the scents deposited by prey species such as house mice are used by predators searching olfactorily, we predicted that 1) significantly more locations treated with prey scents would be visited by a predator than unscented locations, and that 2) locations treated with prey scents would be visited sooner than unscented locations. We used patches of mouse scents to test this prediction in the mallee wheatlands of southeastern Australia. This area supports both house mice and a number of predators thought to hunt using olfaction, including a native predator, the brown snake (*Pseudonaja textilis*), and also introduced mammalian feral predators such as cats (*Felis catus*) and foxes (*Vulpes vulpes*). Dingoes (*Canis lupus dingo*) and their introduced counterpart, the dog (*Canis lupus familiaris*), may also use olfaction to hunt mice.

## Results

**Figure 1:**
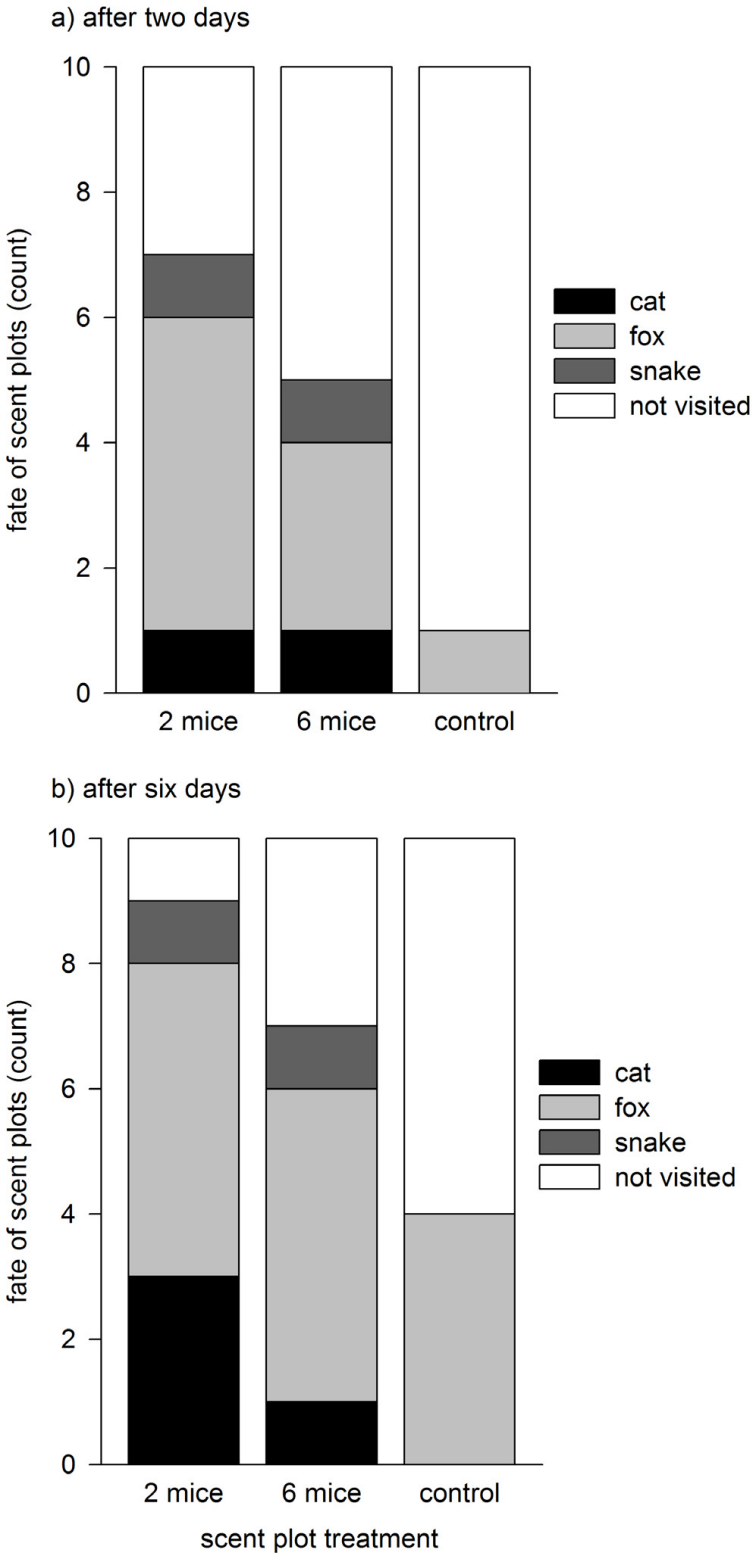
Predators were rapidly attracted to scented plots and visited significantly more mouse-scented plots than control-scented plots. Predators (cats, foxes, and brown snakes) had visited 50% or more of two- or six-mice-scented plots after just two days (Figure 1a). After six days (Figure 1b), the proportion of scent plots treated with the scents of two- or six-mice that were visited by predators was significantly greater than the proportion of control (clean sand) plots that were visited.

**Figure 2:**
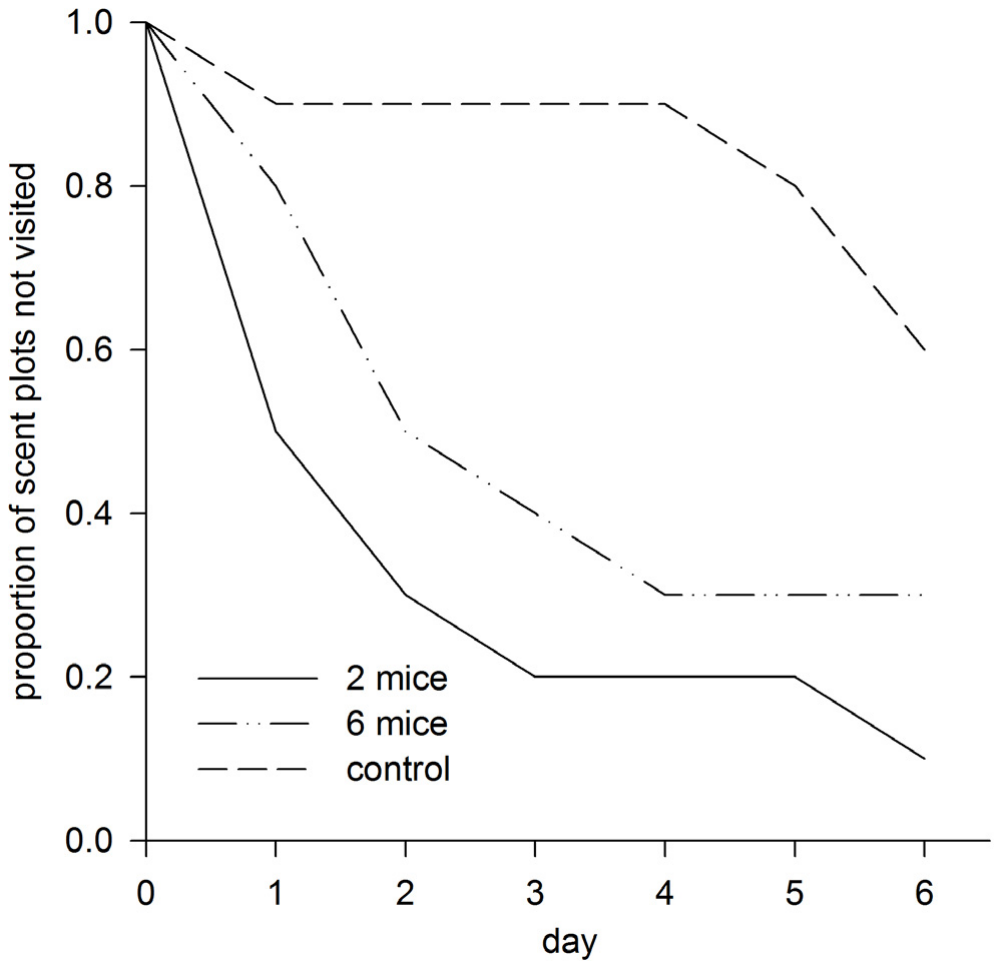
Predators visited mouse-scented plots significantly faster than control-scented plots. Survival analyses revealed that predator visitation rates to the scents of two-mice (solid line) and six-mice (dotted line) were significantly faster than visitation rates to control scents (dashed line). Results are presented as the proportion of plots remaining not visited over time in each treatment.

Cats, foxes and snakes were rapidly attracted to scent plots, visiting more than 50% of scented locations within just two days (Fig. 1a). In contrast, only one control plot had been visited after two days (Fig. 1a; mouse-scented>control: two-tailed Fishers exact test *p* = 0.01). Overall, predators visited significantly more mouse-scented locations than control-scented locations (two-tailed Fishers exact test *p* = 0.04; Fig. 1b). Patterns of scent plot visitation also differed significantly across treatments (χ^2^ = 8.66, d.f. = 2, *p* = 0.01; Fig. 2). The scents of both two and six mice were visited significantly sooner than were plots of clean sand (two mice: χ^2^ = 8.08, d.f. = 1, *p*<0.01; six mice: χ^2^ = 4.74, d.f. = 1, *p* = 0.03). There was no difference in the patterns of predator visitation to scents from two or six mice (χ^2^ = 1.18, d.f. = 1, *p* = 0.28). No dingo/dog prints were detected on plots, however they are likely to be at low densities in the study area due to the low abundance of preferred prey (e.g. rabbits). Consecutive plots were never visited on the same day, and plots were therefore assumed to be independent of one another.

Scent plots were also inspected for mouse prints: none were detected. Although mice will typically be attracted to conspecific odours, this finding was not unexpected as an extended drought had contributed to very low population densities of mice in remnant vegetation habitats (<1 mouse per 100 trap nights).

## Discussion

These results provide the first definitive evidence of predator attraction to prey scent signals in the field. The predators' rapid detection of prey scents suggests that, like the acoustic and visual signals studied so far, the use of olfactory signals will expose individuals to predator mediated selection pressures. Importantly, however, it is not only the signalling individuals that will be at risk of predation from eavesdroppers. All individuals will be at an increased risk of predation when receiving a signal, irrespective of whether they are typically classified as “signallers” or “receivers”. Although dominant males are the primary signallers in many olfactorily communicating species, including mice, most individuals inspect scent marks, some frequently. This intimate association of both signallers and receivers with predator-attracting scent signals therefore suggests that in olfactory signalling systems, a wider range of individuals will be at an increased risk of predation when receiving signals than for other signalling modes [16]. The implications of such a shift in selective pressure to the evolution of signalling systems of different modalities, however, remain largely unexplored.

Olfactory cues are more difficult to conceal than visual or auditory cues because they are long lasting and are necessarily focal points of activity of potential prey. Our findings suggest prey could adopt a variety of strategies to reduce the risks of predator attraction to patches of scent signals. Firstly, prey could distribute their scents to ensure concentrations at patches are below a predator's minimum threshold of detection. Alternatively, prey that perceive an elevated risk of predation could disperse their scent marks away from discrete patches, de-valuing scent patches as cues to predictable prey behaviour. This strategy would, however, necessitate an increase in prey activity when the risk of predation is greatest. As scents are preferentially concentrated around valued resources or boundaries where the likelihood of intrusion is greatest, these strategies are expected to have substantial consequences for resource and territory defence. Finally, prey could alter their activity in relation to scent signals to reduce their risk of predation [14]. Adult male mice, for example, alter the spatial distribution of their activity under an increased level of perceived predation risk, such that movement is minimised, while still maintaining receiving rates of conspecific social signals [19]. However, as little is known of the scale of predator attraction to prey signals, or the scales at which prey detect and respond to predation risk [but see 19], our findings open the way for significant future research.

## Materials

### Ethics statement

All work was conducted in accordance with *The Australian Code of Practice for the Care and Use of Animals for Scientific Purposes (1997)*, and with the approval of the University of New South Wales Animal Care and Ethics Committee (Permit Number 05/98A).

We established a large-scale experiment in the mallee wheatlands of northwestern Victoria, Australia (35°07'S, 142°01'E) during November 2007. This landscape is dominated by large fields (each approximately 4 km^2^); areas of remnant *Eucalyptus* woodland are largely confined to roadsides and nature reserves. Mice are the only small mammal found throughout much of this highly modified landscape, and the diets of foxes [20], cats [21] and snakes [22] therefore contain a high proportion of house mice. Dingoes also consume mice, though they preferentially predate on larger species (e.g. rabbits) [23].

### Scent collection

We trapped adult, male mice (>12 g) around buildings on surrounding farms and housed them in familiar groups of two or three. We held mice in standard mouse cages (48×26×15 cm), with a local sand substrate and shredded paper for bedding, and fed them an *ad libitum* diet of rodent pellets, sunflower seeds, and water.

We established two mouse-scent treatments by collecting the scents produced by groups of two mice and of six mice over a 24 hour period. This number of individuals is representative of the range of mice known to visit a single burrow within 24 hours within this landscape [24]. We used the sand substrate within mouse cages to collect scents from captive mice; mice rapidly scent mark clean substrates [25] and readily deposit scent-marks on sand [16]. Sand collected from exposed areas (unlikely to be visited by mice) in the local area was evenly distributed among mouse cages so that the volume of sand (1 L) used was equal in both odour concentration treatments. The sand substrate was changed every 24 hours and any paper bedding or food particles were carefully removed. The sand therefore contained the mouse scent signals produced within a 24-hour period, but not bedding odours. Mice had been held for 2–4 weeks before odours were first collected; odours collected from conspecifics housed in this way and over this length of time elicited normal investigative and countermarking responses from conspecifics [19]. Cages were sprayed with 70% ethanol and wiped clean before new sand was added. The paper bedding was not changed in order to maintain familiar olfactory cues [26].

### Scent plots

We established scent plots in remnant vegetation beside roads within a study area of approximately 80 km^2^. We selected roads based on the presence of roadside vegetation, a sandy substrate, patchy herbaceous vegetation and infrequent use. Cats and foxes are also known to use roads when moving through these landscapes; plots were at least 500 m apart to maintain independence [27], though several kilometres separated some plots when there was an absence of suitable vegetation and substrates. Each scent plot consisted of a raked area approximately 1.5 m in diameter and located at least 2 m from the side of the road. Scent plots were randomly allocated to one of three treatments (n = 10 per treatment): the scents of two mice, the scents of six mice, and a procedural control, comprising 1 L of clean sand, to control for the addition of sand (hereafter ‘clean sand’). Scented or control sand was added to the centre of each plot, and then smoothed.

Plots were checked every 24 hours and tracks or scats were recorded. Tracks were identified to the species level using species-specific characteristics [28]. Snake tracks were assumed to be those of brown snakes due to their large size [29]. All tracks on scent plots were recorded but a plot was only considered ‘visited’ if it contained the tracks of a predator (fox, cat, or snake). Visited plots were removed from further treatment; plots could therefore be visited only once. Plots that had not been visited were re-treated and the sand surface smoothed. The same mice supplied the scent to a particular scent plot throughout the experiment. We maintained scent plots for up to six days.

### Statistical analyses

We used a contingency table to test whether more mouse-scented plots (two mice and six mice pooled) were visited than control-scented plots after two days and also by the end of the sampling period. We also used Kaplan-Meier survival analyses with log rank tests to examine differences in the time to visitation of scent plots receiving different treatments. We classified scent plots as right-censored if they were discontinued before the maximum time. All analyses were run in JMP7 (SAS Institute).
